# Relationship between pulmonary exacerbations and daily physical activity in adults with cystic fibrosis

**DOI:** 10.1186/s12890-015-0151-7

**Published:** 2015-12-01

**Authors:** Daniela Savi, Nicholas Simmonds, Marcello Di Paolo, Serena Quattrucci, Paolo Palange, Winston Banya, Nicholas S. Hopkinson, Diana Bilton

**Affiliations:** Department of Pediatrics and Pediatric Neurology, Cystic Fibrosis Center, Sapienza University of Rome, Viale Regina Elena, 324-00185 Rome, Italy; Department of Cystic Fibrosis, Royal Brompton Hospital and Imperial College, London, SW3 6NP UK; NIHR Respiratory BRU, Royal Brompton Hospital NHS Foundation Trust, London, SW3 6NP UK; Department of Public Health and Infectious Diseases, Sapienza University of Rome, 00185 Rome, Italy; Eleonora Lorrillard-Spencer Cenci Foundation, 00185 Rome, Italy

**Keywords:** Daily physical activity, Pulmonary exacerbation rates, Cystic fibrosis, Gender difference

## Abstract

**Background:**

The aim of this study was to examine the relationship between pulmonary exacerbations and physical activity (PA) in adults with cystic fibrosis (CF).

**Methods:**

We grouped adults with CF according to their exacerbation status in the year before study enrolment: (1) <1 exacerbation/year; (2) 1–2 exacerbations/year; and (3) >2 exacerbations/year. PA was assessed objectively by means of an accelerometer at the time of study enrolment.

**Results:**

Patients with >2 exacerbations/year spent less time in PA; specifically, fewer activities of mild intensity [>3 metabolic equivalents (METs)], and lower active energy expenditure (*P* = 0.01 and *P* = 0.03, respectively). After correcting for relevant confounders, PA levels were not related to the exacerbation frequency in the preceding year. PA at moderate intensity (4.8–7.2 METs) or greater (>7.2 METs) was independently associated with gender and FEV_1_ % predicted (*P* = 0.007 and *P* = 0.04, respectively). Compared with men, women had reduced vigorous activities (*P* = 0.01) and active energy expenditure (*P* = 0.01).

**Conclusions:**

Adult CF patients with more pulmonary exacerbations in the preceding year have more advanced disease and are less active than their peers. PA was independently associated with gender and airflow obstruction. Gender differences in PA are evident in CF adults.

## Background

Despite many new therapies emerging in cystic fibrosis (CF) [1], lung disease is still the major source of morbidity and mortality for patients with CF [2]. One of the hallmarks of CF lung disease is the recurrence of pulmonary exacerbations that are associated with mortality risk [3], skeletal muscle weakness [4], reduced quality of life [5], and higher health care costs [6]. Many authors have examined the effect of pulmonary exacerbations on lung function [7, 8] and have demonstrated that up to one-third of CF patients will not recover their baseline forced expiratory volume in 1 s (FEV_1_) [7]. Recently, a large group of CF young adults were followed for ~7 years, and half of the decline in FEV_1_ was associated with severe pulmonary exacerbations requiring hospitalisation and intravenous antibiotics [9]. The authors agreed with previous studies confirming that three exacerbations per year represent a crucial tipping point in terms of lung function decline in CF [9, 10]. De Boer et al*.* also demonstrated that CF patients with >2 exacerbations/year have an increased 3-year risk of death or lung transplantation [10].

There is now general agreement among CF health professionals that physical activity improves sputum clearance [11], pulmonary function [12] and muscle strength [13], and it may influence inflammatory markers and immune cell function [14]. There is also evidence about the physiological effects of exercise and the mechanisms by which exercise may improve important clinical outcomes in CF, such as exercise tolerance [15, 16], bone mineral density, CF-related diabetes, and quality of life [17].

Several studies involving CF patients have monitored physical activity during acute pulmonary exacerbation. They have shown that activity levels are lower in patients with acute pulmonary exacerbation compared with stable controls [13], and that energy expenditure is reduced during hospitalisation for respiratory exacerbation compared with 1 month post-discharge [4, 18]. To date, the relationship between pulmonary exacerbation rate and regular physical activity has not been well established in clinically stable adults with CF. No study has sought to understand the impact of frequency of pulmonary exacerbations over 1 year on physical activity levels. The possibility that more frequent pulmonary exacerbations may lead to reduced regular physical activity is worthy of further investigation.

Therefore, the purpose of this study was twofold: (1) to examine the relationship between the number of pulmonary exacerbations in the preceding year and physical activity levels in a clinically stable adult CF population; and (2) compare clinical characteristics and habitual physical activity in adult men and women with CF.

## Methods

### Study design

A cohort of 60 adult patients with CF was recruited at the Policlinico Umberto I Hospital, Sapienza University of Rome, Italy and at the Royal Brompton Hospital, London, UK. Data on pulmonary exacerbations in the preceding 12-month period, demographic and clinical characteristics, and daily physical activity levels were collected at the time of enrolment. The study was approved by the Ethics Committee of Policlinico Umberto I Hospital, with approval number 582/11, and Royal Brompton Hospital, protocol number 07/H0711/125.

### Patients and data collection

Adult patients attending a CF outpatient clinic were approached for participation in the study between January 2012 and May 2014. Patients were included if they were ≥18 years of age and had a confirmed diagnosis of CF based on genetic testing showing two CF-causing mutations and/or two documented sweet chloride values >60 mEq/L. Patients were excluded if they had experienced a pulmonary exacerbation within 4 weeks of the study period; were on the waiting list for lung transplantation; or had undergone lung transplantation. Baseline data were collected at the time of study entry.

After obtaining written informed consent and appropriate screening of medical history, we collected data on age, sex, height, weight, body mass index (BMI), chronic infections and CF comorbidity (pancreas insufficiency and CF-related diabetes). We also collected information on number of pulmonary exacerbations and exacerbation treatment in the preceding 12-month period. Exacerbation events were ascertained through a review of electronic records and hospital charts. To check if there were exacerbations that had not been captured in the health records, direct patient interview was also conducted. Specifically, we collected data on the number of pulmonary exacerbations in the past year requiring oral or intravenous antibiotics. Even if pulmonary exacerbation remains a subjective diagnosis, and unifying definition is still lacking within the CF literature [19, 20], pulmonary exacerbations were defined as acute or subacute worsening of respiratory symptoms, severe enough to warrant oral or intravenous antibiotics. Antibiotic treatment was at the discretion of the treating physician. All patients underwent pulmonary function testing at the time of study entry, which was performed according to American Thoracic Society (ATS) standards, and expressed as percentages of predicted values [21].

### Assessment of daily physical activity

Physical activity was assessed at the time of the study enrolment, using a multi-sensor armband (SenseWear Pro3 Armband; BodyMedia, Pittsburgh, PA, USA) that the patients wore for at least 5 full consecutive days when they were at home. Physical activity data were recorded for 5 full days for all patients and reported as the average of 5 days.

The device was positioned on the upper right arm over the triceps muscle at the midpoint between the acromion and olecranon processes. Patients were instructed to wear the armband day and night and only to remove it for bathing or showering. Patients were also asked to continue any respiratory medications and any of their normal activities. The sensor contained a biaxial accelerometer, galvanic skin response sensor, heat flux sensor, skin temperature sensor, and a near-body ambient temperature sensor from which the data were stored every minute. Using specific software (version 6.1), these variables, as well as body weight, height, handedness and smoking status (smoker or non-smoker), were used to estimate energy expenditure (EE). Several aspects of EE, including total energy expenditure (TEE) and active energy expenditure (AEE), were also calculated. The intensity of physical activity, expressed in metabolic equivalents (METs), total physical activity duration, the number of steps, time lying down, and sleep duration were also measured. The outputs obtained from the armband were time spent in physical activity at different intensities, and the definitions for activity levels based on METs were those used by Troosters et al*.* [16]. The time spent with an EE of 3–4.8 METs was considered mild activity (e.g., walking at normal speed, or carrying out light household work); time spent at 4.8–7.2 METs was considered moderate activity (e.g., brisk walking or cycling); and activities with EE >7.2 METs were considered vigorous (e.g., running or activity with training effects when applied for a sufficient length of time and at an appropriate training frequency) [22]. Studies performed in patients with CF have validated the use of the SenseWear Armband and have shown that the hypersalinity of sweat does not affect the accuracy of EE estimation, and have demonstrated that the device provides an accurate estimate of physical activity in the free-living environment [23, 24]. Finally, it was reported in CF that 5 days monitoring was enough to assess habitual PA and that PA levels were similar through the week (i.e., weekdays versus weekend days) [15, 16].

### Statistical analysis

Baseline characteristics of the group were obtained on the day of enrolment. Patients were grouped according to their exacerbation status in the preceding year: (1) <1 exacerbation/year; (2) 1–2 exacerbations/year; and (3) >2 exacerbations/year. The groupings were made *a priori* before commencing study analysis, based on data in the literature [10]. We selected three exacerbation groups to determine if there was different daily activity related to pulmonary exacerbation frequency. Once established, the exacerbation groupings were used as predefined thresholds and subsequent analysis were performed using these thresholds.

Categorical data are presented as percentages, and comparisons were performed using the *χ*^2^ or Fisher’s exact test. Male and female patients were compared using an unpaired *t* test.

Parametric data are presented as mean ± standard deviation (SD) and comparisons were made using the two-sample independent *t* test or one-way analysis of variance (ANOVA). Non-parametric data are presented as median and interquartile range and comparisons were performed using the Mann–Whitney or Kruskal–Wallis test.

Physical activity variables, age, sex, BMI, FEV_1_ expressed as percentage of predicted (FEV_1_% predicted), genotype, CF comorbidity (diabetes and pancreatic insufficiency) and infection with *Pseudomonas aeruginosa* were compared among pulmonary exacerbation groups, using statistical parametric and non-parametric tests and a multivariate model (ANOVA).

All statistical tests were two-sided, and significance was reported at *P* < 0.05.

## Results

### Study population characteristics and daily physical activity

Baseline characteristics and pulmonary function data for the group as a whole are shown in Table 1. A mean of 2.38 exacerbations per patient was reported: 2.92 in women versus 2.0 in men, *P* = 0.13. Men were older than women and had lower FEV_1_ % predicted (*P* = 0.04), while women tended to have more exacerbations/year. Habitual activity levels were significantly lower in women than men (Table 2). Compared with men, women had reduced TEE in daily life (*P* < 0.0001) and AEE (*P* = 0.01) and lower average METs (*P* < 0.05). Activities with vigorous intensity were lower in women (*P* = 0.01). Similarly, there was a trend for less activity at moderate intensity [women 10 (6–24) min vs 19 (9–33) min for men; *P* = 0.16) and less duration of physical activity (women 184 ± 115 min vs 233 ± 149 min for men, *P* = 0.17). After adjusting for other covariates, these physical activity variables remained significantly higher among male patients.

**Table 1:** Anthropometric characteristics and pulmonary function of 60 patients with CF

Characteristics	CF (*n* = 60)	Males (*n* = 35)	Females (*n* = 25)
Age, yr	33.5 ± 10.5	36.3 ± 11.0*	29.6 ± 7.6
BMI, Kg/ m^2^	23.1 ± 3.0	23.2 ± 2.7	23 ± 3.4
FEV_1_,L	2.59 ± 0.79	2.74 ± 0.88	2.37 ± 0.59
FEV_1_, % predicted	73.1 ± 19.9	68.8 ± 20.9*	71.9 ± 16.8
FVC, L	3.80 ± 1.00	4.22 ± 1.08*	3.22 ± 0.54
FVC, %predicted	90.0 ± 18.0	88.0 ± 21.7	93.3 ± 12.2
TLC, % predicted	98.5 ± 18.8	93.9 ± 19.8*	105.0 ± 15.0
DL_CO_, % predicted	83.9 ± 15.5	84.6 ± 17.1	82.9 ± 13.3
*Pseudomonas aeruginosa* colonization, n (%)	39 (65)	24 (68.5)	15 (60)
*Staphylococcus aureus* colonization, n (%)	41 (68.3)	26 (74.2)	15 (60)
*Burkholderia cepacia* colonization, n (%)	2 (3.3)	2 (5.7)	0 (0)
Pancreatic insufficiency, n (%)	40 (66.6)	23 (65.7)	17 (68)
CF-related diabetes, n (%)	14 (23.3)	8 (22.9)	6 (24)
508 del homozygous/heterozygous	14/33	6/23*	8/10
Number of exacerbations/year	2.38 ± 2.36	2.00 ± 1.69	2.92 ± 3.00

Data are presented as mean ± SD, unless otherwise stated

*DL*_*CO*_ diffusion capacity of the lung for carbon monoxide; *FVC* forced vital capacity; *TLC* total lung capacity

*P < 0.05, differences between men and women with CF

**Table 2:** Daily physical activities measured by the accelerometer in 60 patients with CF

Variable	CF (*n* = 60)	Males (*n* = 35)	Females (*n* = 25)
Total energy expenditure, kcal	2710 ± 592	2957 (2542–3217)*	2309 (2114–2524)
Active Energy expenditure, kcal	899 ± 602	830 (508–1562)*	617 (385–856)
Steps, number/day	9508 ± 3861	9984 ± 4246	8842 ± 3213
Duration Physical Activity, min/day	213 ± 137	233 ± 149	184 ± 115
Average METs	1.7 ± 0.3	1.8 ± 0.3*	1.6 ± 0.2
Mild intensity activities, min/day	186 ± 121	199 ± 129	168 ± 110
Moderate intensity activities, min/day	15 (9–29)	19 (9–33)	10 (6–24)
Vigorous intensity activities, min/day	1 (0–3)	1 (0–5) *	0 (0–0)

Data are presented as mean ± SD or median and interquartile range, unless otherwise stated

*P < 0.05, differences between men and women with CF

### Association between pulmonary exacerbation and physical activity

Eleven patients had <1 exacerbation/year, 31 had 1–2 exacerbations/years, and 18 had >2 exacerbations/year (Table 3). The group with more frequent exacerbations had a higher proportion of patients with a severe genotype (*P* = 0.001), pancreatic insufficiency (*P* = 0.006), CF-related diabetes (*P* = 0.006), and had chronic *P. aeruginosa* infection (*P* = 0.008). Patients who experienced >2 exacerbations/year had lower baseline FEV_1_; both expressed as absolute values and % of predicted (*P* = 0.01 and *P* = 0.05, respectively). As shown in Table 3, compared with patients who had <1 or 1–2 exacerbations/year, the group with >2 exacerbations/year spent less time in daily physical activity (*P* = 0.01). Daily activities at mild intensity were markedly reduced in patients with >2 exacerbations/year (*P* = 0.01). AEE (i.e., number of calories per day due to physical activity) and average METs were lower in the group with more frequent exacerbations (*P* = 0.03 and *P* = 0.01, respectively). Conversely, there was no significant difference for activities with at least moderate and vigorous intensity among the three groups (Table 3).

**Table 3:** Demographic characteristics of the whole group of 60 patients based on the exacerbation frequency

	<1Exacerbation/year *n* = 11	1-2 Exacerbations/year *n* = 31	>2 Exacerbations/year *n* = 18	Between-group *p* value
Male/Female	7/4	19/12	9/9	0.68
Age, yr	32.5 ± 11.9	34.2 ± 10.2	33 ± 9.7	0.86
BMI, Kg/ m^2^	23.3 ± 3.4	22.6 ± 2.8	23.9 ± 3.1	0.33
508 del homozygous/heterozygous	0/10	8/16	6/7	0.001
Pancreatic insufficiency, n (%)	3 (27.2)	22 (70.9)	15 (83.3)	0.006
CF-related diabetes, n (%)	1 (9.1)	4 (12.9)	9 (50)	0.006
Lung function				
FEV_1_,L	3.09 ± 0.77	2.6 ± 0.73	2.25 ± 0.75	0.01
FEV_1_, % predicted	85 ± 22	72.5 ± 16	66.9 ± 21.8	0.05
FVC, L	4.2 ± 1.1	3.8 ± 0.97	3.4 ± 0.9	0.14
FVC, %predicted	97.3 ± 21.7	89.8 ± 16.2	86.5 ± 19.7	0.31
Chronic infections, n (%)				
*Pseudomonas aeruginosa*	3 (27.2)	21 (67.7)	15 (83.3)	0.008
*Staphylococcus aureus*	9 (81.8)	24 (77.4)	9 (50)	0.1
*Burkholderia cepacia*	0 (0)	1 (3.2)	1 (5.5)	1
Daily physical activity				
Total energy expenditure, kcal	2701.3 ± 706.7	2793.2 ± 631.9	2570.6 ± 428.4	0.45
Active energy expenditure, kcal	638 (349–699)	1003 (550–1377)	606.5 (464–752)	0.03
Steps, number/day	9449 ± 4981	10394 ± 3923	8018 ± 2492	0.11
Duration physical activity, min/day	142 (80–225)	262 (159–327)	135.5 (85–182)	0.01
Average METs	1.6 (1.5–1.9)	1.8 (1.6–2.1)	1.6 (1.5–1.8)	0.01
Mild intensity activities, min/day	126 (72–219)	213 (127–310)	119.5 (81–167)	0.01
Moderate intensity activities, min/day	9 (5–21)	19 (9–38)	14.5 (6–26)	0.3
Vigorous intensity activities, min/day	1 (0–2)	0 (0–4)	1 (0–2)	0.88

Data are presented as mean ± SD or median and interquartile range, unless otherwise stated

There was however, no association between the number of pulmonary exacerbations in the preceding year and physical activity variables when corrected for clinical covariates (age, sex, BMI, FEV_1_% predicted, infection with *P. aeruginosa*, genotype, diabetes and pancreatic insufficiency). Daily physical activity was independently associated with gender and airflow obstruction. TEE and AEE were related to sex (*P* < 0.0001 and *P* = 0.01, respectively). Physical activities above the threshold of moderate (4.8–7.2 METs) and vigorous (>7.2 METs) intensity were also related to sex and FEV_1_% predicted (*P* = 0.007 and *P =* 0.04, respectively). Finally, the number of steps was lower in those with a lower FEV_1_% predicted, although this was not significant (*P* = 0.09).

## Discussion

This study suggests that adult CF patients with more pulmonary exacerbations in the preceding year have more advanced disease and are less active than their peers. After correcting for lung function and anthropometric variables, there was no independent relationship between pulmonary exacerbation frequency and daily physical activities. In the present study, the level of physical activity was independently related to gender and airflow obstruction (FEV_1_% predicted). This indicates that other factors rather than pulmonary exacerbation rate contribute to the reduced habitual physical activity in a period of clinical stability. Our results confirm that adult women with CF are significantly less active than their male counterparts and they tend to have more exacerbations per year.

Unfortunately, despite developments in CF management, pulmonary exacerbations continue to be one of the hallmarks of CF lung disease, and the annual prevalence of pulmonary exacerbations increases with age [2]. Our study shows for the first time that patients with >2 exacerbations in the preceding year have a lifestyle characterised by lower daily physical activity (including activities with mild, moderate and vigorous intensity) and lower AEE compared with patients with fewer exacerbations. This is also the first population-based cohort study showing that physical activity at mild intensity level (e.g., walking at normal speed or shopping, and carrying out light household work) is significantly reduced among patients with frequent exacerbation. Moreover, we found that patients with frequent pulmonary exacerbations in the preceding year were more likely to have diabetes, infection with *P. aeruginosa*, and have lower lung function. This is not surprising, and our data are in line with other studies in which patients with frequent exacerbation were diabetic, female and had more rapid decline in FEV_1_ from baseline compared with those with infrequent exacerbation [10]. Diabetes, *P. aeruginosa* infection and reduced FEV_1_ are known risk factors for pulmonary exacerbations in CF [25]. The novelty of our study is that patients with >2 exacerbations in the past year also have reduced daily physical activity. Even with a prospective study design, it is difficult to infer causality and whether the inactivity aggravated the decline of FEV_1_, inhibited diabetes control, and trigged more exacerbations. In order to assess causality, the effect of interventions aimed at enhancing habitual physical activity should be studied.

Patients with <1 exacerbation/year had less comorbidity and better lung function but were more sedentary compared with those with 1–2 exacerbations/year for all the parameters measured by the accelerometer. Specifically, regarding physical activity levels at moderate intensity, the CF group with <1 exacerbation/year was less active than those with more exacerbations. Duration of moderate activities was 9 min in patients with <1 exacerbation/year, 19 min in those with 1–2 exacerbations/year and 14.5 min in those with >2 exacerbations/year. It is possible that the differences in the moderate activity results reflect motivation to participate in the study; are related to sampling error (i.e., small number of patients); or related to greater encouragement to exercise as a consequence of more frequent healthcare interaction.

Having adjusted for baseline clinical covariates, we observed that there was no independent relationship between the number of pulmonary exacerbations in the previous year and physical activity variables. Any conclusion about causal links between exacerbations and habitual physical activity must therefore be limited. The two most important determinants of physical activity variability in the present study were gender and FEV_1_% predicted.

We found that daily activity at moderate intensity or greater and EE were independently associated with sex, with higher values among male patients. When gender was evaluated separately, adult women with CF were significantly less active than their male counterparts, even when adjusted for previously mentioned covariates. Although some physical activity parameters only tended to be higher among men, these results still have significant clinical relevance. Similar gender differences in habitual physical activity have previously been shown in a paediatric CF population [26, 27]. Our study is the first to demonstrate that this carries over to adulthood, using an objective measurement. We have also noted a trend for more exacerbations per year in women than in men with CF.

It may be important to identify new strategies to improve physical activity among women with CF who do not voluntarily engage in their habitual daily life. Further research is needed to determine whether strategies for promotion of activity work equally for both sexes. There is clearly a need to study a different approach to stimulate physical activity among women, considering that gender differences in habitual activity are evident after the onset of puberty [27]. Recently, Cox et al*.* [28] have emphasised the lack of evidence regarding the most effective strategies to promote uptake and continued participation in physical activity for CF patients. We believe that activities of mild intensity (e.g., walking around the home or office, shopping, and walking from the parking lot) are simple behaviours that could be easily advised at any medical encounter with CF patients and incorporated into any management plan.

It is of note that a multivariate analysis identified FEV_1_% predicted as an independent, statistically significant predictor of physical activity above moderate intensity. This would suggest that in stable CF patients, high activity levels in daily life do reflect the level of lung function. This is in line with previous observations that aerobic performance is correlated with measures of lung function [16, 29]. Our study underlines that, in this clinically stable CF population, the time spent doing activities of moderate intensity or greater is positively related to pulmonary function.

The study had some important limitations. First, we showed that CF patients with >2 pulmonary exacerbations in the past year were more sedentary compared with those with less frequent exacerbations. Even with a prospective study design for physical activity monitoring, it is not possible to infer direct causality from these observations, and whether less physical activity occurred prior to the identification of the exacerbations or was a direct result of several exacerbations. In fact, a request to conduct prospectively planned cohort studies to provide a stronger evidence base for determinants and outcomes of physical activity was recently made to the research community [30]. Interventional studies should test the efficacy and effectiveness of physical activity programmes in reducing the individual annual rate of pulmonary exacerbations in CF. The second limitation was the retrospective nature of the data collection for pulmonary exacerbation rates, which may have underestimated the rate of exacerbations. Even if our CF patients were closely followed up and exacerbation events were ascertained through a review of electronic medical records and hospital charts, some mild exacerbations may not have been recorded. Finally, there was the definition of exacerbations. A single agreed definition of pulmonary exacerbations is lacking [19, 20] and pulmonary exacerbations defined by change in treatment (i.e., new oral or intravenous antibiotics) provides a pragmatic definition used in other publications [9, 10].

## Conclusions

Adults with CF who had >2 pulmonary exacerbations in the year prior to physical exercise assessment had more advanced disease and were less active than patients with 1–2 or < 1 exacerbation/year. Specifically, daily activities at mild intensity were markedly reduced in patients with frequent CF exacerbations. After correcting for relevant confounders, the level of daily physical activity was not related to the exacerbation frequency in the preceding year, but it was independently associated with gender and pulmonary obstruction. Gender differences in daily physical activity are evident in adult patients with CF. This study adds to the mounting evidence that, in clinical practice, we should recommend the benefits of keeping an active lifestyle that does not require huge effort and can be incorporated into daily life easily, especially for patients who are known to have frequent exacerbations. These findings strengthen the evidence to support the development of different strategies to promote regular physical exercise in women with CF. More research is needed in CF to investigate the efficacy of exercise or physical activity interventions on definitive clinical endpoints such as exacerbation rates and survival.

## Abbreviations

PA: Physical activity
METs: Metabolic equivalents
FEV_1_: Forced expiratory volume in one second
